# Clinical placement models for undergraduate health professions students: a scoping review

**DOI:** 10.1186/s12909-021-03023-w

**Published:** 2021-12-04

**Authors:** Champion N. Nyoni, Lizemari Hugo-Van Dyk, Yvonne Botma

**Affiliations:** School of Nursing, Faculty of Health Sciences, University of the Free State, Bloemfontein, South Africa

**Keywords:** Clinical placement, Models, Health professions education, Undergraduate, Scoping review

## Abstract

**Background:**

Clinical learning is fundamental to undergraduate health professions students. There are several calls for the transformation of health professions education, which have direct implications on clinical learning. Clinical placement models provide structure to clinical learning. Therefore, this scoping review could contribute to supporting curriculum transformation to enhance learning in the clinical environments for undergraduate health professions students.

**Objectives:**

This scoping review identified the characteristics of research evidence related to mapping the purpose, methodologies used, outcomes, and specific recommendations associated with clinical placement models in undergraduate health professions education.

**Design:**

A scoping review method was used in this study. A search string developed from the title of the review was used to search online databases to identify research published between January 2000 and March 2020.

**Results:**

Forty-eight articles reporting on ten clinical placement models were included in this review. The majority of these articles originated from Australia and predominantly report on nursing. The aims of these articles aligned with the evaluation of the implementation of a clinical placement model. Seven categories of outcomes of the clinical placement models are reported namely, relationships, influence, environment, facilitation, inputs, knowledge scores, and student perceptions.

**Conclusions:**

As clinical learning is fundamental to undergraduate health professions education, clinical placement models should prioritise the development of competence among undergraduate students. Insights into outcomes reported in literature could guide educators in fostering optimal learning in students who may then be able to influence community health outcomes positively.

## Background

The last decade saw various calls for innovation in undergraduate health professions education. Frenk et al. [1], in their seminal article, support the adoption of transformative education, while the World Health Organization (WHO), in the Framework for Action on Interprofessional Education and Collaborative Practice [2], argues for the infusion of interprofessional education in undergraduate health professions programmes. The Carnegie Foundation for the Advancement of Teaching highlights the need for reforms in health professions educations, which foster the development of learning environments that integrate classroom and clinical concepts emphasising clinical reasoning [3]. More recently, the State of the World Nursing report has reaffirmed the need for competency-based education (WHO, [4]). These recommendations to undergraduate health professions education are made against a backdrop of increased student numbers, resource limitations, healthcare system challenges and emerging health crises, such as the COVID-19 pandemic, inevitably implying that traditional clinical placement models may not support learning [5].

Clinical placement models are a theoretical structure that guides educators and health professional students in their engagement with authentic clinical opportunities [5]. This theoretical structure integrates the purpose of the placement, the placement activity, the location of the placement – including the length of placement, students, supervision and placement facilitators [6]. Clinical placements are critical in health professions for the application of learnt clinical skills in authentic settings. Clinical placement models that increase placement capacity are cost-effective and ensure a positive learning culture, are critical for competence development [7]. Innovations in undergraduate health professions education programmes must integrate relevant clinical placement models into their mainstream education programmes.

The calls for a transformation in health professions education merge with theoretical shifts that foster the adoption of active education models as an alternative to passive teacher-centred models [8]. Active education models are oriented towards learning, shifting the education responsibility to the student, who is expected to place himself or herself at the centre of the education process [9]. Constructivism, as an educational theory, advances active learning, and has been reported in underpinning the design and development of undergraduate health professions education programmes in some countries [10]. In such programmes, the students create meaning by connecting their ideas with their experiences, both inside the classroom and out. Clinical placement models should enable undergraduate health professions students to transfer their learning into practice and be aligned with active learning models.

At the time of this study, the authors could not find a comprehensive review of post-2000 literature on clinical placement models in undergraduate health professions education. The aim of this article is to report on a scoping review that sought to answer the question “What is known from the literature regarding clinical placement models for undergraduate health professions students?” A mapping of the literature on clinical placement models used in undergraduate health professions education could contribute to supporting programme directors, clinical educators and curriculum planners in fostering optimal learning for students in the clinical environment.

## Methods

### Scoping review method

The scoping review method as described by Peters et al. 2020 (cited in Aromatis & Munn, 2020) [11] was used. Scoping reviews are an approach to synthesising knowledge that addresses an exploratory research question aimed at mapping key concepts, evidence characteristics, recommendations and gaps in a specific research area [11, 12]. Aromatis and Munn [11] and the Preferred Report Items for Systematic and Meta-analysis extension for Scoping review (PRISMA-ScR) as described by Tricco et al. [13] influenced the conceptualisation, execution and reporting of this scoping review. This scoping review was executed through several steps namely the search strategy which included the search string, the information sources and the inclusion criteria. Additional steps were; a search for the evidence, selection of evidence, analysis of the evidence, and presentation of the results. The Preferred Reporting Items for Systematic Reviews and Meta-Analysis Protocols (PRISMA-P) guided the development of a priori protocol [14]. The Health Sciences Ethics Committee (HSREC) of the University of the Free State (UFS) approved the protocol (HSD 2020/0572/2605), registered at the Open Science Framework (See https://osf.io/8e463).

### The search string

**Fig. 1:**
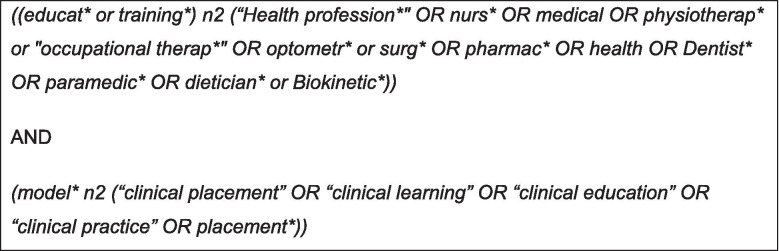
Search string

The Medical Subject Headings (MeSH) keywords influenced the generation of the search string based on the key concepts of the title. Through discussion between the authors and a university librarian, and secondary to several trials searches, the final search string is presented in Fig. 1.

### Information sources

The following electronic bibliographic databases were searched, namely CINAHL with Full Text, MEDLINE with Full Text, Academic Search Ultimate, APA PsycInfo, Health Source, Nursing/Academic Edition, ERIC, Africa-Wide Information, Open Dissertations, and APA PsycArticles. Additional sources of information through an ancestry search after examining the reference lists of articles included in the final search supplemented the literature.

### Inclusion criteria

This review sought to include literature regarding clinical placement models used in undergraduate health professions education. Peer-reviewed research articles were included if they were published between January 2000 and April 2020 and available in English. Studies applying different research designs inclusive of qualitative, quantitative and mixed methods research were included in this review. However, grey literature, reviews and studies referring to clinical teaching or facilitation, which only referred to an aspect of clinical placement model, were excluded.

### Selection of source of evidence

**Fig. 2:**
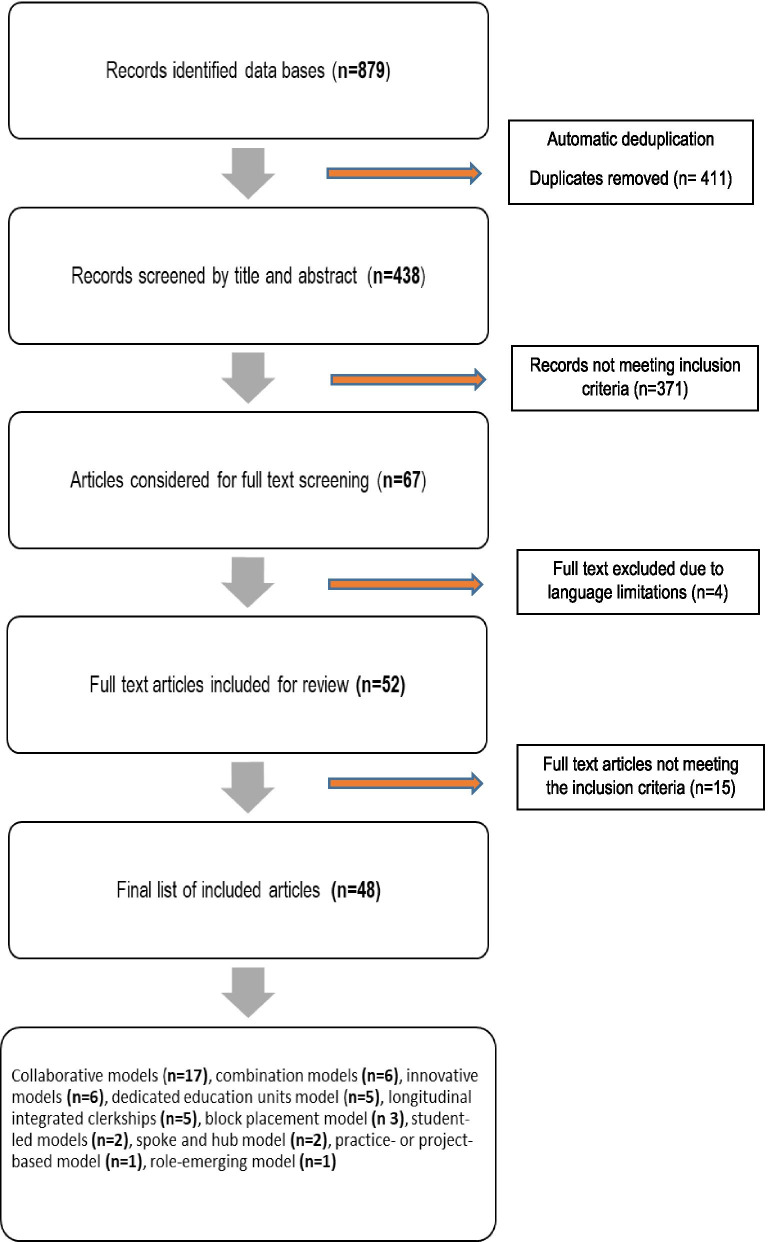
Data selection process

The initial search produced 879 hits and after automatic de-duplication resulted in 438 hits for the first round of screening. The authors independently screened and selected the evidence, meetings were held to discuss the findings of the individual screening. Discrepancies among the authors were resolved through discussion. An initial examination of titles and abstracts against the inclusion criteria eliminated 371 records that did not meet the inclusion criteria. Through a university librarian, full-text articles were sought and screened against the same inclusion criteria. Fifteen full-text articles did not meet the inclusion criteria and were eliminated. A further four full-text articles were eliminated as they were not in English, and translation efforts were futile (see Fig. 2).

### Charting the data

The authors jointly developed a data charting form. The data charting form was independently piloted on two included articles by the authors, and discussions enhanced its refinement. Characteristics of the articles, the professions, the research design, and the purpose of the research, data collection methods, specific outcomes, and recommendations from the included studies were charted. One author charted this data on an electronic tool supported by the ATLAS.ti platform, and the other two authors independently verified the accuracy of the data. Discussions resolved inconsistencies and amended some missing data (see Table 1).

**Table 1:** Summary of data charting

Author and year	Country	Profession	Design	Name of placement model	Purpose of the study	Outcomes	Recommendations
McDonnel Smedts and Louw*. (*2008) [15]	Australia	Medicine	Quantitative	Block	To investigate the association of the placement approach with return to practice in the placement environment	- there was an association between longer periods of placement and the return of graduates to take up internship in the Northern Territory	- research on the elements of longer placements and students who return - investigation on the characteristics of students that return
Birks et al. (2017) [16]	Australia	Nursing	Qualitative	Block	To compare students’ perceptions on the impact of block placement over distributed placements	- “We’re there to learn” - block has advantage of consistency over distributed placement - feeling part of a team	- adequate preparation of clinical environment and the staff who support students undertaking clinical placements - distributed placements may be of greater value earlier in the programme and block placements in senior years - ensure appropriate sequencing of placements to align with theoretical and chronological stages of study - promoting consistency by enabling students to return to a familiar venue - adequately preparing the clinical environment for students they receive on placement - establishing a culture that encourages students to feel as though they are members of the team
Kell and Owen (2009) [17]	United Kingdom	Physiotherapy	Case study	Block	To describe the possible influence of the placement learning environment on students’ approaches to learning	- the numbers of educators and assessors involved in the placement education affect the approach students adopt to their learning. - having more than two students in any one placement may increase their fear of failing the placement - students achieving the highest placement grades are those who perceive themselves to be strategic learners with a very low fear of failure - students with the lowest placement marks have the highest fear of failure - deep learning may be greatest on lone student placements - surface approaches to learning may be greatest where two students share a placement - students perceived the greatest ability to monitor their effectiveness on individual placement - for most students, there was a strong positive relation between predicted and actual placement scores - students with the greatest difference between their self-rated and actual placement marks were those who perceived themselves as deep learners - there was no gender interaction	none reported
Blakely et al. (2009 )[18]	United Kingdom	Occupational Therapy	Not reported	Collaborative *2:1 placement model*	To describe the students’ experience of the 2:1 placement model	- individual students offer a confidential arena to discuss issues - peer supervision allowed for students to learn about different learning styles - combining supervision styles was helpful for students - model worked well where there was no need for a clinical case load - an opportunity to enable students to engage in a range of supervision styles	- need for more clinical facilitators
Cohen et al. (2015) [7]	United States	Midwifery	Quantitative	Collaborative *clinical learning dyad model (CLDM)*	To evaluate the CLDM model through a survey to assess usage, attitudes and perceived programme benefits and challenges	- increased programme capacity - increased interaction between students - opportunity for peer-to-peer learning - scheduling flexibility benefit - less time doing orientation	- research on the impact of the model on senior students
Kreulen et al. (2008) [19]	United States	Nursing	Quantitative	Collaborative *clinical education partnership model*	To evaluate the clinical education partnership model	- students were satisfied with the model - students met course objectives, understood special health care needs, and felt comfortable intervening to help children and families - staff agreed that students helped in meeting health needs of the community	- commitment of both the school district and college administration is crucial - leaders from each partnership agency are needed - positive ongoing interaction
Newton et al. (2012) [20]	Australia	Nursing	Quantitative	Collaborative *preceptor partnership model*	To investigate the clinical learning environment from the perspective of students participating in a structured partnership model	- students did not rate the clinical learning environment favourably - student-centeredness was reported from the students the structured model - continuity of the clinical teacher is integral in facilitating students’ engagement in the learning environment	- research on the tensions between clinical practice environments and student learning is recommended - follow-up with both levels of practitioner is essential
Newton et al. (2011) [21]	Australia	Nursing	Qualitative	Collaborative *clinical partnership model*	To describe factors that facilitate or hinder nursing students’ perceptions of their work-readiness upon graduation	- students felt support and were made to feel part of the professional team - students acknowledged understanding staff and organisation, and not having to ‘re-learn’ information about each setting afforded them greater confidence and the ability to participate meaningfully - the work environment fostered a sense of belongingness and generated a degree of work-relatedness	- the model is dependent on the commitment of nurses - recommendations for preceptor training and recognition - further research on alternative partnership models of clinical learning to ensure that graduates ‘hit the ground running’ - research on how healthcare organisations could foster a culture of learning practice
Bhagwat et al. (2018) [22]	Australia	Speech and language therapy	Mixed methods	Collaborative *paired and single clinical placement models*	To compare paired and single clinical placement models relative to time use by students and clinical educators	- Clinical educators and student time use was not significantly different when comparing placement models - the clinical educators paired and single cohorts had similar time use - high satisfaction with both models	- paired placements can be used with limited implication on service and time - clinical educators and students need engagement in patient- and non-patient-related activities
Lynam et al. (2015) [23]	Ireland	Dietetics	Qualitative	Collaborative *2:1 placement model*	To pilot the 2:1 placement model	- a framework for implementing a 2:1 model was developed	- need for optimal guidelines - the need to schedule one-on-one time within the 2:1 model to enhance transparency
Nash et al. (2009) [24]	Australia	Nursing	Mixed methods	Collaborative *transition model*	To describe the preparedness to practice by final-year nursing students exposed to two separate clinical placement models	- there was no significant difference between students exposed to the collaborative model and those who were not regarding their preparedness - a sense of belonging to a team - being part of a team - experiencing the real world of nursing practice - students in the trial model rated themselves higher in preparedness before and after the intervention	- essential to match students to an appropriate placement model - students should be aware of the placement options - further research on student characteristics and matching with transitional models, including their preferred learning styles
Martin et al. (2004) [25]	United Kingdom	Occupational Therapy	Qualitative	Collaborative *1:1; 2:1; 3:1 placement model*	To determine the impact of different models of practice placement (1:1, 2:1 & 3:1) on the quality of education for students and on the quality of the experience for educators in Occupational Therapy	- a general preference on the 2:1 model - peer support in the 2:1 & 3:1 clinical placements - peer learning opportunities - time for facilitating learning in the 1:1 model was more - essential for adequate planning in all the models	- practice educators need to familiarise themselves with new approaches to peer learning - higher education institutions should continue to inform students and practice educators of the benefits of being associated with multiple placement opportunities - design authentic placement opportunities informed by the environment - research on different models for placement models for different settings
Alpine et al. (2019) [26]	Ireland	Physiotherapy	Quantitative	Collaborative *2:1 model*	To evaluate practice placements using 2:1 supervision and implementation models by students and practice educators	- shared learning experience was identified as a benefit of the model - peer support environment - the development of peer evaluation and feedback skills by students	- it is essential to match students with appropriate clinical placement models - clear guidance to students on the provision of peer feedback and support for educators in providing feedback to two different students
Moore et al. (2003) [27]	United Kingdom	Physiotherapy	Qualitative	Collaborative 1:1, 2:1 and 3:1 placement models	To compare experiences of clinical educators and students using different clinical placement models	- success of a placement model is dependent on planning - all placement models have a place in physiotherapy education although there was a general preference for the 2:1 placement models	- it is essential that placements be well planned - enough time should be made available for the educator to meet individual students - sufficient patients are made available for students
Price and Whiteside (2016) [28]	Australia	Occupational Therapy	Qualitative	Collaborative *2:1 model*	To examine supervisor experience in a trial of the new model, namely the 2:1 model	- strategies, such as consulting evidence, being prepared, setting joint tasks, seeking support, and adopting a positive attitude	- supervisors should seek out research and practitioner knowledge and guidance - supervisors should plan and identify learning opportunities in advance - individual supervision should be made available for students - organisational and collegial support should be mobilised and formalised - ensure patients are comfortable with student numbers - communication between students and supervisors - positivity and pragmatism
O’Connor et al. (2012) [29]	Ireland	Occupational Therapy	Qualitative	Collaborative *2:1 and 1:1 placement models*	To describe the perspectives of clinical educators and students who participated in both the 1:1 and 2:1 models of clinical education across Occupational Therapy and Physiotherapy	- learning experience from the 2:1 models was better than from the 1:1 model - 1:1 model were easier and better to organise compared to the 2:1 model - relationships enhanced the function of the models, between supervisors and also among students (2:1 model)	- further research may be warranted to explore the timing of different clinical education models - need for different types of preparation per model - need to increase the emphasis on formative assessment - essence of preplanning to enhance the implementation of the model - educators need to be aware of individual students’ learning needs to avoid student dissatisfaction
Barnett et al. (2010) [30]	Australia	Nursing	Qualitative	Collaborative *university and rural hospital partnership*	To develop and evaluate a collaborative model for clinical education that would increase the capacity healthcare agency to accommodate student placements and improve workplace readiness	- the model resulted in 58% increase in the number of students and 45% increase in capacity of placements over a a four-year period - students valued access to and working one on one with preceptors - staff was approachable	- there is a need to test models of preceptorship that could build the capacity of a hospital when there are fewer full-time and more part-time RNs available as preceptors
Didion et al. (2013) [31]	United States	Nursing	No design reported	Collaborative *integrated clinical education model*	To describe the implementation of a clinical teaching model	none described	- strong partnership between the university and a larger integrated healthcare environment
Taylor et al. (2015) [32]	Australia	Nursing	Multiple methods	Collaborative The Hospital and University Learning Investment Project (*TULIP) model*	To describe students’ perceptions, preceptors’ understanding and organisations’ partnership capacity and capability over a three-year period	- staff and students ready to learn together because of collaboration and multi-pronged collaboration - partnership approach - increase placement capacity	none reported
Svejda et al. (2012) [33]	United States	Nursing	Qualitative	Collaborative *partnership model*	To describe the pilot outcomes of implementation of a partnership model	- enthusiasm in participating in pilots	- need to adapt the model in the changing healthcare environments
Gilmour et al. (2013) [34]	Australia	Midwifery	Qualitative	Combination b*lock and integrated 2 days per week*	To compare students’ experiences between two undergraduate models of clinical placement	- student role - facilitated learning - belonging to the team - no one model was favoured, the role of bad midwives influenced students’ experiences	none reported
Greenhill et al. (2018) [35]	Australia	Medicine	Qualitative	Combination b*lock,* *Longitudinal Integrated Clerkship,* *Community and hospital-integrated learning*	To determine the transformations students experienced within the sociocultural context of clinical practice	- self-awareness - patient-centredness - systems thinking - self-care - clinical scepticism - understanding diversity from seeing people	- medical schools must include the learning objectives of critical thinking, clinical scepticism and system thinking in the clinical curriculum to promote a perspective change in their graduates
Patterson et al. (2017) [36]	Australia	Nursing	Quantitative	Combination *traditional multi-facility clinical placement model (TMFCPM), the university fellowship programme (UFP) and the mixed programme (MP)*	To examine perceptions of work-readiness of new graduate nurses who attended one of the following three clinical placement models: - traditional multi-facility clinical model (TMFCPM) - the university fellowship programme (UFP) - the mixed programme (MP)	- only the university fellowship programme was significantly associated with better work-readiness - students’ understanding of and integration with an organisation were better when transitioning to the graduate nurse role	- organisational partnerships enhance graduates’ work-readiness
DeMeester et al. (2017) [37]	United States	Nursing	Qualitative	Combination *traditional learning models (TLM), hospital learning model (HLM) practice–education partnership (PEP)*	To gain insight into the experiences of students, preceptors and staff member stakeholders in three clinical learning models: the traditional learning models, the hospital learning model and the practice-education partnership	- promotion of learning - hindrances to learning - preceptors’ perceptions of positive aspects - preceptors’ recommendations for change - staff perceptions of most and least effective aspects of clinical models - inclusion of nurse, authenticity and non-comprehension	- further study to understand the findings related to the practice of clinical education better and more broadly
Henderson et al. (2006) [38]	Australia	Nursing	Quantitative	Combination *facilitation model; preceptor model and the clinical education unit*	To assess the perceptions of undergraduate nurses of the psychosocial characteristics of clinical learning environments within three different clinical placement models	-the preceptor model rated high on all measures of clinical learning inventory - the facilitation and the clinical education unit yielded high scores on student numbers	- models required to be adapted to suit particular university curricula and clinical environments
Kumm et al. (2016) [39]	United States	Nursing	Quantitative	Combination models *Models not specified*	To compare the perceptions of nurse preceptors of students’ proficiency during clinical immersion models	- no significant difference between immersion models	- competition for clinical sites as well as time and cost to support lengthy rotation offerings should be considered
Goslee et al. (2020) [40]	United States	Nursing	Multiple methods	Dedicated Education Units	To assess the perception of nursing students and clinical teachers of the quality of the dedicated education units clinical education model in primary care	- positive experiences with the DEU model - learning needs were a priority due to continued communication and collaboration between staff nurses and staff members	none reported
Grealish et al. (2018) [41]	Australia	Nursing	Qualitative	Dedicated Education Units	To determine the feasibility of a clinical placement model	- feasibility is dependent upon aligning stakeholder expectation with the new model, clarify roles and responsibilities within clusters, develop strategies for collecting information about student performance	- clarification of roles and responsibilities to be continually negotiated - further research into the model, its overall effectiveness and efficiency, as well as further research to find elements that are critical for implementation - more research on how students, newly qualified nurses and staff learn in localised work units and practice pedagogies in practice
Hannon et al. (2012) [42]	United States	Nursing	Qualitative	Dedicated Education Units	To describe the experiences of nursing students when partnering with the same registered nurse throughout the clinical experience	- relationship building - communication was essential between staff and university based educators	- adjustment of the timetables to enable student function
Mulready-Schick et al. (2013) [43]	United States	Nursing	Quantitative	Dedicated Education Units	To describe the effect of the dedicated education units in education quality in nursing students	- education quality and learning gains are significantly more positive for students clinically instructed in dedicated education units - positive learning experience by students - greater growth in clinical learning and development of nursing knowledge - more time spent on instructional activities	- focused research on evaluation of clinical performance using standardised tools
Moscato et al. *(*2013) [44]	United States	Nursing	Qualitative	Dedicated Education Units	To describe the replication of the dedicated education units at two universities in the United States	well replicated	none reported
McKenna et al. (2009) [45]	Australia	Midwifery	Qualitative	Innovative model *home hospital clinical placement model*	To explore health professional students’ clinical experiences and their impact on career intentions	- enhance familiarity with clinical staff - increased knowledge retention	- students should undertake clinical placements in the same clinical setting for an extended period of time; - further research in other professions and contexts should be done on benefits of bringing clinical experiences directly into the classroom setting to extend learning
Timm et al. (2021) [46]	United States	Multi-professionals (*Nursing, Exercise Sciences & Graduate Counsellor Education*)	Mixed method	Innovative model *interprofessional faculty–student-led clinical education model*	Evaluate how a new clinical education model, focused on interprofessional practice and service to the community, would provide clinical training for nursing and other education programmes effectively	- interprofessional growth and teamwork, unorthodox learning environment - a culture of ‘team-ness’ - primary and secondary prevention, reaching underserved in the community,	- Investigation of strategies to enhance the faculty–student-led clinics, curricular development, and inform other programmes.
Mumford (2007) [47]	United Kingdom	Medicine	No method stated	Innovative model *clinical academies*	Evaluation of an innovative clinical placement model	- no deterioration in standards of student achievement - positive rating from students - students applauded the organisation and leadership	- universities to adopt this placement model
Kevin et al. (2010) [48]	Australia	Nursing	Multiple methods	Innovative model *alternative weekly placements*	Explore students’ experiences of an innovative model	- students learnt and felt supervised - integration of learning - sense of familiarity - lack of time for clinical learning	- recognise the value of the sense of familiarity among students and the clinical staff
Fairbrother et al. (2016) [49]	Australia	Physiotherapy	Mixed methods	Innovative model *capacity development facilitator (CDF) model*	Evaluation of a pilot of the capacity development facilitator model through the identification of enablers, barriers and stressors in the clinical placement	- learning resources and support were identified as superior - another set of ‘eyes and ears’ on site made the students feel comfortable - stress levels were higher at the beginning of placement compared to the end, and students reported the support from the work and capacity development facilitator model helped them to manage stressors - placement allocation data increased in student placement capacity ranging from 63 to 153%	- the introduction of the capacity development facilitator model and its evaluation in different contexts - further research to ensure the cultivated, mutually beneficial relationships, improved student learning outcomes and enhanced services for consumers are maintained
Marshall et al. (2012) [50]	United Kingdom	Nursing	Mixed methods	Innovative model- ‘*the sandwich approach to clinical placement’*	To examine the implementation of an initiative that enabled third-year adult field student nurses the opportunity to spend the final year of education within the community setting	- confidence - placement diversity - knowledge, skills and diversity	- ensure the skills students are expected to achieve would cover those needed for the community setting
Campbell et al. (2019) [51]	Australia	Medicine	Quantitative	Longitudinal Integrated Clerkships	To compare first, the work locations (regional or more rural) of medical students, following registration as a medical practitioner, who had other types of rural training of comparable duration elsewhere, and second, students who had no rural training	- students who undertake the East Gippsland rural longitudinal integrated curriculum and additionally participate in rural training in other years (average rural training during 2.0 years) are the most likely group to subsequently work in smaller regional or rural towns - students whose only rural training was through Longitudinal Integrated Clerkship platforms were more likely to be in rural practice than those who only trained in metropolitan areas - the study revealed that medical students who undertake rural training for more than a year (non-Longitudinal Integrated Clerkships) were more likely to work rurally than students wholly training in metropolitan areas	-strong and influential locally representative leadership is a prerequisite for sustaining rural Longitudinal Integrated Clerkships programmes
Poncelet et al. *(*2014) [52]	United States	Medicine	Quantitative	Longitudinal Integrated Clerkships	To compare knowledge scores between students engaged in a LIC and those who were not	- scores at entering clerkship did not differ between the students - clinical knowledge scores did not differ at the completion of the clerkship - the clinical performance examination scores demonstrated significantly higher performance by Longitudinal Integrated Clerkship students in the data gathering domain - the perceptions of the Longitudinal Integrated Clerkship students did not differ significantly from the traditional students - Longitudinal Integrated Clerkships students received significantly higher internal medicine examination scores that traditional students	- communication and coordination - contribution from experienced administrators - partnerships could focus on longitudinal clinical experiences - systems-based practice or population health experiences and curricula in a functional primary care system
Daly et al. (2013) [53]	Australia	Medicine	Qualitative	Longitudinal Integrated Clerkships	To illustrate the pedagogical and socio-cultural underpinnings of student learning theoretically within a longitudinal, integrated, community-engaged rural placement	- the physical and social geography shaped the scope and nature of student learning and socialisation experiences - the placement comprised a series of interconnected learning spaces - the variability in supervision was shown to limit student learning and active participation - connectivity was a key process by which the boundaries between these learning spaces were identified, negotiated and crossed	- students can be coached to recognise the embedded Community of Practice and social networks - medical educators need to work on developing reciprocal relationships - it is also important to ensure that students receive practical guidance
Ogur et al. (2007) [54]	United States	Medicine	Multiple methods	Longitudinal Integrated Clerkships	Evaluate the Harvard Medical School- Cambridge Integrated Clerkship (HMS-CIC) clinical placement model	- 100% of the HMS-CIC students responded that “very often” or “often” they saw patients before diagnosis and decision for admission, compared with 20% of comparison group. - 100% of the HMS-CIC students responded that they very often or often saw patients they treated after discharge - students received feedback often and mentoring from faculty when compared to other students - Objective Structured Clinical Examinations scores were similar and higher in some areas when compared to other students - OSCE revealed that the HMS-CIC students had higher scores for communication. HMS-CIC students found the year rewarding when compared to the other students	leadership and support and ongoing faculty development programme
Boardman et al. (2019) [55]	Australia	Nursing	Qualitative	Longitudinal integrated clerkships	To evaluate the perspectives of students and preceptors of the mental health integrated clinical learning model in an Australian metropolitan acute admission and extended care rehabilitation unit	- preparedness for practice - maintaining a work–life balance - perceived being part of the team protracted learning	none reported
Hunt (2006) [56]	Australia	Occupational Therapy	No method stated	Project-based clinical placement models	Describe the project-based model and assessment outcomes	- raised profile of the profession - autonomy and responsibility allow students to gain important professional and generic skills - provision of an aspect of service that had previously been out of reach - high level of satisfaction by students	- exploration of student experiences of project-based placement
Linnane and Warren (2017) [57]	Ireland	Occupational Therapy	Quantitative	Role-emerging	To generate views from occupational therapists and occupational students on the use of role-emerging placements in the Republic of Ireland	- wider gains of community - promoting profession - opportunity to transfer knowledge - poor communication - difficulty in assessment of students	- research on organisational culture, rigour and vigilance for learning
Roxburgh et al. (2012) [58]	United Kingdom	Nursing	Multiple-case study	Spoke and hub	To develop, implement and evaluate the impact of spoke and hub models of practice learning across geographically diverse locations	- belongingness - different learning experience - adaptability of skills at hub - communication and teamwork - logistical changes	-improved communication at hub
Thomas and Westwood (2016) [59]	United Kingdom	Nursing	Qualitative	Spoke and hub	To describe students’ experiences related to the spoke and hub model	- belongingness as a pair of hands to being part of a team - broader understanding of patients’ journey, context, connection and continued learning - mentors are helpful and enthusiastic - some mentors need orientation to the placement model	-improved communication between hub and spoke
Grealish et al. *(*2013) [60]	Australia	Nursing	Mixed methods	Student-led	To determine the viability of (student-led placement) innovation by developing a preliminary understanding of what students were learning and exploring stakeholder perceptions about student learning	- knowledge scores increased after placement - students reported deep learning - enactment of complex concept e.g. professional identity and self-realisation, collegiality, willingness to share, responsibility, accountability	- designing the placement should have adequate infrastructure for large numbers of students - further research in organisational learning culture where students are placed
O’Connor et al. (2019) [61]	Ireland	Physiotherapy	Qualitative	Student-led	To explore the challenges and facilitators of a community-based, student-led placement involving physiotherapy undergraduate students and community-based groups	- leadership, teamwork and ability to accommodate individual service user needs - sense of anxiety regarding placement structure prior to commencement - mutual and shared learning experiences - challenges in embracing change	- continued engagement of service users in the design and implementation of the placement model

### Analysis of data

Multiple steps were applied in analysing the charted data. In the initial step, the authors examined the characteristics of the included articles, namely the year of publication, the country where the research was conducted, the profession and the research design. Frequencies in each of these characteristics were tallied. The subsequent step examined the outcomes and recommendations of the research studies included in this review. In this step, the authors inductively thematised the outcomes of the studies to generate 19 broad themes influenced by the review question. The included articles were then clustered based on the reported clinical placement model and appraised against the 19 broad themes. Furthermore, the themes were combined into seven descriptive categories.

## Results

The results of this review are presented through a discussion of the characteristics of the included studies and the outcomes of the models.

### Characteristics of the included studies

Forty-eight full-text articles were included in this scoping review. The included articles comprised a range of studies from a 20 year period and were from Australia (*n* = 22), the United States (*n* = 13), the United Kingdom (*n* = 8) and the Republic of Ireland (*n* = 5). Seven professional disciplines were represented, namely Nursing (*n* = 25), Medicine (*n* = 7), Occupational Therapy (*n* = 6), Physiotherapy (n = 5), Midwifery (*n* = 3), Dietetics (*n* = 1) and Speech and Language Therapy (n = 1). Qualitative research (*n* = 19) was the predominant design used in most of the included studies followed by quantitative designs (*n* = 13), then mixed methods (*n* = 6), multiple methods (*n* = 4), non-specified designs (*n* = 4), and case study research (*n* = 2). Ten clinical placement models used for undergraduate health professions students were reported, namely, collaborative models (*n* = 17; 7, 18-33), combination of models (*n* = 6; 34-39), innovative models (*n* = 6; 45-50), dedicated education units (*n* = 5; 40-44), longitudinal integrated clerkship placement model (*n* = 5; 51-55), block placement model (*n* = 3, 15-17), student-led placement model (*n* = 2; 60-61), spoke and hub placement model (*n* = 2; 58-59), practice- or project-based placement model (*n* = 1; 56) and the role-emerging placement model (*n* = 1; 57) (see Table 2).

**Table 2:** Brief explanation of the clinical placement models

CLINICAL PLACEMENT MODEL	BRIEF EXPLANATION
Block	Block placement models integrate full-time clinical placements for periods of weeks within study periods or vacation breaks. Apprenticeship style of learning underpins block placement models. Students have to achieve specific objectives which are aligned to the placement site [16].
Collaborative	Collaborative placement models emphasise on the assignment of two or more students to a clinical facilitator namely, 1:1; 2:1; and 3:1 placement [25]. In this review, articles that further include clinical learning dyads, preceptor partnerships were included under collaborative placement models.
Combination	Some studies reported a combination of established clinical placement models to enhance specific outcomes. Combination models in this review, reflect an amalgamation of established clinical placement models [37].
Dedicated Education Unit	The dedicated education unit are models that reflect specific units or wards within a hospitals that are dedicated for the clinical placement of students [41].
Innovative models	Innovative models for clinical placements reflects non-conventional models for clinical placement that were perceived as new by the implementers in their setting such as clinical academies, alternative weekly placements, capacity development facility models and sandwich approaches [45–50]
Longitudinal Integrated Clerkships	Longitudinal integrated clerkships reflected a situations where students are engaged in comprehensive patient care for extended periods of up to a year, through relationships with clinicians aimed at acquiring core clinical competences across disciplines simultaneously [52].
Practice or project based	Practice or project based models encompassed community based placement approaches where students collaborated with the community in analysing, planning, implementing and evaluating participatory community practice projects [56].
Role- emerging	Role-emerging placements take place in non-conventional settings without the expected specific health professional employed, with supervision offered by onsite employees and also distant support from the health professional concerned [57]. For example placing Occupational therapy students in environments were Occupational therapists do not ordinarily work, and the students being supported through distance approaches by Occupational therapists from their University [57].
Spoke and hub	In spoke and hub models, students are allocate to a ‘hub’ for specific practice, and are further allocated to spoke placement which is associated with the speciality of the hub with an idea of enhancing understanding of the journey of the patient through healthcare setting [59].
Student led	Students lead service provision within their settings under the guidance of their clinical facilitators [61]

### Outcomes of the models

Seven categories, including 19 themes, were inductively generated after engaging with the outcomes and recommendations of the included articles. The outcomes of the studies were mapped against the 19 inductively generated themes and seven categories. The majority of the themes from the review were reported among collaboration models (see Table 3).

**Table 3:** Summary of the outcomes

	OUTCOMES OF CLINICAL PLACEMENT MODELS	ROLE EMERGING	SPOKE AND HUB	STUDENT-LED	PROJECT-BASED	INNOVATIVE	BLOCK	LIC^a^	COMBINA-TION	COLLABORA-TION	DEU^b^
**RELATIONSHIPS**	Belonging to a team		✓			✓	✓	✓	✓	✓	
	Peer support among students			✓						✓	
	Helpful/Positive relationships between the clinical teacher and students		✓					✓	✓	✓	✓
**INFLUENCE**	Benefiting the community	✓			✓	✓			✓	✓	
	Professional image promotion	✓		✓	✓				✓		
	Influence of placement model on career outlook						✓	✓	✓		
**ENVIRONMENT**	Consistency of placement and continuing patient care		✓			✓	✓	✓			
	Diversity of the placement environment					✓		✓	✓		
	Increased placement capacity									✓	
**FACILITATION**	Facilitation time and flexibility in placements									✓	
	Knowledge transfer opportunity	✓	✓		✓	✓	✓	✓			✓
	Students receiving feedback	✓						✓			
**INPUTS**	Need for adequate resource planning		✓			✓				✓	✓
	Need for orientation to the clinical placement model		✓								
**KNOWLEDGE SCORES**	No effect on clinical knowledge scores					✓		✓	✓	✓	
	Improved knowledge scores			✓		✓		✓			
**STUDENT PERCEPTIONS**	Student perceived learning		✓	✓						✓	
	Student satisfaction and positive experiences			✓	✓	✓				✓	✓
	Student self-efficacy associated with improved outcomes			✓			✓			✓	

^a^*LIC* Longitudinal integrated clerkships

^b^*DEU* Dedicated Education Units

## Discussion

The purpose of this scoping review was to describe what is known on clinical placement models used in undergraduate health professions education. The majority of the included articles were from Australia and a significant number were drawn from other high-income countries, namely the United Kingdom and the United States. According to Plancikova et al. [62], these high-income countries generally have more funding for research and resources to conduct research when compared to low-income countries predominantly in Africa and Asia. As only studies in English were included, studies from non-English-speaking contexts related to clinical placement models in undergraduate health professions education were not considered. Hence, this review was skewed towards high-income English-speaking countries.

Evidence generated from undergraduate nursing dominated the number of articles included in this review, even though Medicine, Occupational Therapy, Physiotherapy, and Midwifery were also found. The literature explains that the distribution patterns of health professionals are skewed towards nursing and medicine, with nursing being the single largest group of health professionals in healthcare [4]. There are limited numbers of other health professionals including the limited number of health professions education institutions providing their training. Programme directors and administrators in Nursing and Medicine often battle with student clinical placements [61]. The large number of students enrolled in undergraduate nursing and medicine against the dwindling clinical placement opportunities in traditional clinical placement platforms may drive some programme directors to be creative in guaranteeing that their students attain professional competencies [7], the creativity can also be true with programmes will smaller student enrolments. Furthermore, professional culture, the context where the placement models is applied including issues such as finance could explain the trends of association between placement models and specific professions.

Specific professions seem to favour one clinical placement model over the other. For example, Medicine and Nursing seemed to report on clinical placement models that accommodate a large number of students, such as block placement models [34] while professions with a smaller number of students, such as occupational therapy, dominated reports on collaborative models that allow for more intimate supervision [22]. These decisions seem to be influenced by the purpose of the placement, the number of students, the placement capacity and availability of supervisors.

This review highlighted that the majority of research in the field of clinical placement models focuses on evaluating outcomes associated with the implementation of clinical placement models. The studies report on the influence of clinical placement models on the experiences of students and educators, and specific measurable outcomes, such as knowledge scores. Only two of the included articles had longitudinal outcomes inclusive of community impact. The Kirkpatrick evaluation model is a popular model for analysing and evaluating results of educational programmes [63]. According to the Kirkpatrick evaluation model, the majority of studies (*n* = 45) included in this review were aligned with the bottom two levels, namely level 1: reaction, and level 2: learning [63]. This signifies a gap in research for studies that evaluate the outcome of such clinical placement models on students, the clinical setting and the community. Longitudinal studies that evaluate the impact or higher levels of the Kirkpatrick evaluation model are therefore needed.

The outcomes of the studies included in this review were grouped into seven main categories, namely relationships, influence, the environment, facilitation, inputs, knowledge scores, and student perceptions. Firstly, relationships were reported as outcomes of specific models, such as belonging to a team [55], peer support among students [26], and helpful or positive relationships [40]. Nordquist et al.*,* [64] explain the importance of positive relationships where students learn from and with their peers and facilitators. Students are reported as being able to learn when they are supported through positive relationships by their facilitators, their peers, and when they have a sense of belonging [59]. The establishment, development and nurturing of positive relationships among students, peers and their supervisors is an essential component of any clinical placement model and clinical placement coordinators should aim towards establishing positive relationships to enhance learning.

The second outcome reported by some of the studies included in this review, was the influence of the clinical placement models on students and the communities within which they worked. The influence of the clinical placement models referred to specific benefits to the community [57], promotion of professional image [56], and even influence on the career paths of students [51]. Evidence from service-learning interventions has shown short- and long-term health influence on communities by student-led health intervention [65]. Student-driven learning cements students’ understanding of the clinical environment and such understanding has been reported to influence career trajectories and promote the image of their profession [10]. However, students within the clinical setting should be supported sufficiently to promote an appropriate professional image while at the same time understanding their own professional remits to enhance their influence in the clinical environment.

The third outcome was the environment, which was reported as a complex multi-faceted structure that could enable or disable student competence development [64]. The physical environment, the patient–condition diversity, the number of students and facilitators, including the availability of resources are aspects of the clinical learning environment [66]. Fundamentally, any clinical placement model in undergraduate health professions education should cultivate a learning environment that enables students to meet their learning outcomes and develop competence. From this review, some of the included articles reflected the environment as part of the outcomes. The studies reported the implementation of specific clinical placement models to increase placement capacity [49], while others reflected on models for enhance consistency of the clinical environment and continued patient care [53]. Greenhill, et al. [35] report that their clinical placement model allowed students an opportunity to access diverse patient conditions.

Fourthly, the outcome of facilitating competence development in the clinical learning environment requires a clinical placement model that allows students to transfer learning in the clinical environment under the supervision of expert clinical educators and for opportunities of continuing feedback [23]. According to Clark [67] learning may be perceived as students integrating new concepts from their already existing knowledge schemas. The student’s prior knowledge needs to be explored, before he or she can assimilate and accommodate new knowledge [68]. According to this review, some clinical placement models provide an opportunity for students to transfer learning from the classroom to the clinical environment [59], while other models are flexible enough to enhance the facilitation of learning within the clinical environment. Daly et al. [53] report enhanced opportunities for student feedback as an outcome of the implementation of their clinical placement model. However, it appears as if models that had a lower supervisor and student ratio report on outcomes related to feedback opportunities, while larger numbers of students limit the opportunities for individualised feedback. Individualised feedback cements the assimilation and construction of new knowledge, especially in a complex clinical environment. However, individualised feedback opportunities may not always be possible in many health settings, especially in low- and middle-income countries that face shortages of the health workforce and educators against increased student numbers [69].

Specific inputs are necessary for the application and implementation of specific clinical placement models. In this review, some of the articles expressed a need for orientation to the clinical placement models for both the students and the clinical staff [59]. Orientation to a clinical placement model is essential for students to meet their expected clinical outcomes and for facilitators who are expected to support learning [70]. Some clinical placement models also require extraordinary resources. In their description of the dedicated education units (DEU), Springer et al. [71] state that they aimed at creating an ‘ideal’ clinical environment to facilitate authentic learning. However, procuring additional resources to create an ideal clinical environment may be a challenge, as additional resources may be impractical in some settings.

The implementation of some of the clinical placement models was evaluated through the examination of the sixth outcome of student knowledge scores after placement. On the one hand, some articles reported an increase in student knowledge scores attributed to the clinical placement model [54] while on the other hand, Poncelet et al. [52] reported no significant changes in student knowledge scores. Knowledge is part of competence when integrated with appropriate skills and attitudes within an authentic clinical environment [72]. Experience in the clinical environment brings to life the theoretical knowledge obtained from the classroom setting, and when valid assessments are applied, learning becomes meaningful [73]. The aim of clinical placement models should go beyond the improvement of knowledge scores towards competence attainment. A clinical placement model that contributes to competence development and attainment could contribute to a competent health workforce that influences health outcomes.

Student perception is the seventh outcome reported in the articles included in this review. Le, et al. [74] note that students are at the centre of their learning, and their perceptions and learning experiences influence the development of their self-efficacy. In the studies reviewed, some student perceived to have learnt from being included in specific clinical placement models [61], and were satisfied with their learning [56]. Nash et al. [24] report improved student self-efficacy associated with a clinical placement model. Liu [75] explains these finding through stating that students’ perception of the clinical learning environment influences their learning and acceptance of important teaching and learning strategies, such as feedback. Grant [76] adds that for feedback to be meaningful and to result in learning students need to have positive perceptions of their mentors and that of learning. A negative perception and experience may increase students’ cognitive load, which may become a barrier to learning [77]. Any clinical placement model used in undergraduate health professional education should foster positive perceptions and experiences, as these supports the development of self-efficacy and competence.

Studies included in this review also reported specific recommendations that should be applied in relation to each clinical placement model. In essence, the studies recommend further research on student characteristics and how such characteristics may be aligned with specific clinical placement models for optimal learning. Further research is proposed on the application of specific models in different contexts and different professions, to establish the influence of clinical placement models on learning. The included studies also recommend the investigation of organisational culture, its link to clinical placement models, and eventually learning. Practical recommendations include –

- planning for the clinical placement, such as preparation of the environment;
- guidelines for model implementation;
- sequencing of placement; and
- learning opportunities as a priority recommendation.

Specific articles emphasised the need and role for communication and coordination between the institutions, the students and the facilitators.

### Strengths and limitations of this review

In establishing the rigor of this review, the authors aligned the review process and decisions on reporting with contemporary frameworks of reporting a scoping review. In addition, throughout the review process, the authors – who are qualified and who possess relevant experience and expertise – worked and made decisions independently but where there were discrepancies, these were resolved through discussion. A university database, accessed with the support of a university librarian generated the data for this review. The possible limitations of this review arose from the search string and inclusion criteria, which may have eliminated some studies. Such studies in non-English languages might have been beneficial and influential in terms of the outcomes of this review.

## Conclusion

This study employed a scoping review methodology guided by contemporary frameworks to describe what is known from the literature regarding clinical placement models in undergraduate health professions education. The majority of these models were reported from Nursing and from Australia, with a paucity in research from low- and middle-income countries. In most of the articles reviewed, the longitudinal effect of the clinical placement models was not reported. In addition, these articles did not describe competency-development models, which align with the purpose of a clinical placement model. Various outcomes were reported as associated with specific clinical placement models, and these outcomes reflected the complexities of the clinical learning environment. Although this review was not aimed at identifying a superior clinical placement model, the outcomes of these studies demonstrate some of the essential aspects of clinical education in undergraduate health professions.

Programme directors, administrators, clinicians and educators in undergraduate health professions education are constantly faced with decisions related to the adoption of clinical placement models that enhance optimal learning influenced by calls for a radical transformation of health professions education. No single clinical placement model exists as the panacea for undergraduate health professions education; however, the context, the educational programme and educational design may be used to influence decisions on adopting a clinical placement model. Based on the insights of this scoping review of literature on clinical placement models we recommend the following essential elements that may be integrated to enhance learning among undergraduate health professions students:

- all models need to prioritise the establishment, development and nurturing of positive relationships between students, peers, and their facilitators;
- students in clinical placements must be supported by experienced professionals;
- students need to be facilitated to meet competence through individualised feedback;
- all clinical placement models need specific orientation and resources; and
- clinical placement models should incorporate the development of positive learning experiences and perceptions from students.

Further longitudinal research could focus on the effect of specific models on students, the clinical environment and community outcomes. As clinical learning is fundamental to undergraduate health professions education, students need to be exposed to clinical environments that enable the development of their competence. Insight into outcomes reported in the literature could guide educators in their quest to transform undergraduate health professions education. In doing this, programme directors, administrators, clinicians and educators could provide innovative programmes that would foster optimal learning for the development of competent health professions graduates who may positively influence health outcomes in many communities.

## Data Availability

All data sets used and/or analysed during the current review are available from the corresponding author on reasonable request.
